# Managing the value Co-creation of peer service providers in the sharing economy: The perspective of customer incivility

**DOI:** 10.1016/j.heliyon.2023.e16820

**Published:** 2023-06-01

**Authors:** Yu Jia, Qinyu Chen, Wenlong Mu, Wei Zhang

**Affiliations:** a School of Journalism and Communication, Wuhan University, 430072 Wuhan, China; b School of Safety Science and Emergency Management, Wuhan University of Technology, Wuhan 430072, China

**Keywords:** Sharing economy, Customer incivility, Value co-creation, Conservation of resources theory

## Abstract

Previous studies have discussed the preconditions for peer service providers participation in value co-creation from the perspective of the platforms or the peer service providers themselves. However, little attention has been paid to the influence of customers. In the sharing economy, however, customers interact closely with peer service providers, and they have a major influence on the attitudes and behaviours of peer service providers. Based on resource conservation theory, this study uses three waves of tracking surveys and two experiments to investigate the impact of customer unfriendliness on the shared value creation of peer service providers. The results suggest that rude customers in the sharing economy reduce the value creation behaviour of peer service providers by increasing emotional exhaustion. Furthermore, self-efficacy in regulating negative emotions is found to buffer the mechanism by which customer incivility increases the likelihood of emotional exhaustion. Peer service providers with higher self-efficacy in negative emotion regulation have weaker such relationships. This research fills the gap of how customers influence the value creation behaviour of peer service providers in the sharing economy, identifies the potential negative impacts of customer incivility and increases the overall added value of the sharing economy.

## 1 Introduction

Currently, the sharing economy has entered a new stage of development in China. In 2021, its transaction scale in China exceeded 3.6 trillion RMB, and the number of participants exceeded 800 million [1]. The sharing economy is based on the Internet and verified through trust. It refers to the interaction between persons or entities to commute benefits or remuneration by transferring the temporary use right or dominium of idle resources [2]. It can be characterized as triadic; specifically, (a) customers seek access to assets, (b) peer service providers (PSPs) offer idle resources, and (c) platforms enable matching and transactions [3]. Both customers and PSPs are users of sharing economy platforms.

Co-creation refers to a process in which customers actively create value with a company [4]. According to Vargo et al. [5], value co-creation occurs when there exists the “integration of resources and application of competencies.” In the sharing economy, value is co-created through collaboration between users and other potential users [6,7]. As a recent innovation in marketing, value co-creation is considered an important driver of green purchasing behaviour [8] and provides new opportunities for sustainable consumption and production [9]. Value co-creation can help firms enhance brand fame, win brand value, and offer new opportunities for organizations to gain competitive advantages and achieve sustainable development [10,11].

Prior research on value co-creation in the sharing economy has focused primarily on the value co-creation of consumers [2,12,13], while limited attention has been given to the value co-creation of PSPs [14]. However, in sharing economy services, the PSPs who provide the services and the customers who use those services form a bilateral market [15]. The value of the platforms to the PSPs is influenced by customers, and the value of the platforms to the customers depends on the PSPs. Therefore, the sharing economy service development strategy obtained by analyzing only the customers or the PSPs is not integral [15]. Because numerous trustworthy service providers are needed to attract customers, the development of sharing economy platforms largely depends on the continuous participation of PSPs [16,17]. Hence, platforms need to comprehend the antecedents of the participation of PSPs in value co-creation.

Several research gaps that deserve attention are identified based on an overview of the extant literature regarding the antecedents of the value co-creation of PSPs in the sharing economy. Previous research on the antecedents of the value co-creation of PSPs has mainly focused on the platforms and PSPs themselves. Some studies have considered the factors of the platforms as the antecedents for the participation of PSPs in the sharing economy [14,[17], [18], [19], [20], [21]], such as via the feedback mechanism, protection mechanism, and dispute resolution mechanism [22]. Others have focused on PSPs themselves [14,[17], [18], [19],^23^,^24^], exploring the impact of internal motivation on their value co-creation [25,26]. However, prior research has given limited attention to the antecedent variables of the value co-creation of PSPs from the perspective of customers. The sharing economy is characterized by synergy, and different participants can influence each other [27,28]. For example, Shokoohyar [21] found that experiencing bad rider behaviour is one factor considered in drivers’ ratings on platforms. Thus, the influence of customers on PSPs cannot be ignored.

In the sharing economy, many PSPs (e.g., ride-share drivers and delivery staff) have faced situations including being despised and belittled or even insulted, abused, provoked, and threatened by customers. These low-intensity deviant behaviours carried out by customers with a vague intent to harm the PSPs reflect customer incivility [29,30]. Examples include condescendingly belittling people, ignoring their statements, and excluding them from social friendships. Prior research has primarily focused on customer incivility toward employees in the workplace, such as by investigating its impact on the extra-role customer service of employees [31]. Nevertheless, in the sharing economy context, the object of customer incivility has changed from company employees to PSPs. Compared with employees, PSPs play an equal role in the relationship with customers, and their participation in the service is always to meet their personal needs [32]. Moreover, the contracts signed between PSPs and platforms have less binding force than those signed between employees and companies. Furthermore, they do not need to be entirely subject to the management activities of the enterprise regarding production and service activities. However, it remains unclear whether the perception of customer incivility by PSPs in the sharing economy will be more sensitive than that of employees in the workplace and whether customer incivility will affect the value co-creation behaviour of PSPs.

Against the backdrop of the sharing economy, to explain why the adverse interaction between customers and PSPs affects the value co-creation of PSPs, customer incivility is examined as an antecedent of the value co-creation behaviour of PSPs. The mediating role of emotional exhaustion is also tested based on the conservation of resources (COR) theory and from the perspective of customer-provider interaction. Furthermore, to explore approaches to the management and control of the inevitable problem of customer incivility in the sharing economy, the moderating role of self-efficacy for negative emotion regulation (SENER) in the association between customer incivility and the value co-creation of PSPs is explored.

**Fig. 1:**
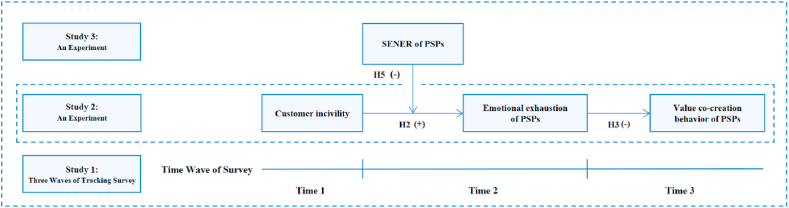
The research model. *Note.* +: positive association, -: negative association. Time 1: 2021.12.19, Time 2: 2022.01.03, Time 3: 2022.01.18. SENER = Self-efficacy for negative emotion regulation, PSPs = Peer service providers.

Via three waves of tracking surveys and two experiments, the impact of customer incivility on the value co-creation of PSPs and the mediating role of emotional exhaustion are examined. Moreover, from the perspective of the individual characteristics of PSPs, the moderating role of SENER in the impact of customer incivility on the value co-creation of PSPs is tested. Our hypotheses are examined by multiple analytical methods, such as OLS regression and ANOVA. Fig. 1 provides a comprehensive summary of the research model and proposed hypotheses.

## 2 Theoretical foundations and hypotheses

### 2.1 Conservation of resources theory

As a motivation theory, the fundamental doctrine of COR theory is that individuals are encouraged to maintain, preserve, and obtain the resources that they regard as valuable [33], such as happiness, family, belongingness, respect, and meaningful life. When resources are lost, unstable, or threatened, or when individuals cannot cultivate and protect their resources through their efforts, pressure will arise [34].

COR theory posits that when threatened by potential or actual resource loss, individuals and groups will actively acquire, retain, cultivate, and protect valuable resources to meet future needs [34]. Individuals with fewer resources are more likely to be affected by resource loss and have a lower ability to obtain resources. Therefore, they tend to maintain existing resources rather than risk the depletion of resources [[35], [36], [37]]. In accordance with the theory, by hurting the emotional and psychological state of PSPs, customer incivility (as a source of stress) exhausts the resources that are needed to complete the service [38] and results in the emotional exhaustion of the PSP. When individuals face the risk of resource depletion, they will reduce the input of existing resources [39] and minimize behaviour that requires spending their remaining resources, such as avoiding value co-creation.

Hence, a logical chain of “customer incivility – emotional exhaustion – reduction of value co-creation behaviour” is constructed. In addition, the belief and ability of PSPs to manage anger or irritation will affect the degree of emotional resource loss. Therefore, SENER is proposed as the boundary condition for this relationship.

### 2.2 Hypotheses

#### 2.2.1 The relationship between customer incivility and value Co-creation of peer service providers

Hobfoll [33] noted that the fundamental doctrine of the COR is that people are motivated to preserve, guard, and acquire resources. People seek to gain resources through resource investment; this is not solely limited to investing working time to obtain economic returns but also refers to the investment of abstract resources (e.g., energy and trust) [36,37]. The authors believe that value co-creation, as a behaviour of investing current resources to obtain possible resources (e.g., appreciation, praise, and positive emotions), is a resource investment of PSPs [36].

In the context of the sharing economy, taking the ride-hailing industry as an example, customer incivility – such as causing trouble after drinking, defacing the vehicle, smoking in the car, taking off their shoes, vigorously closing the door, carrying pets without communication in advance, making loud calls when carpooling, and asking for the driver's personal information – occurs from time to time. These behaviours consume the central resources required for accomplishing services and introduce the risk of resource depletion to PSPs [38].

According to COR theory, individuals experience more significant distress and anxiety when faced with resource loss. Thus, they are motivated to guard and preserve resources [33,34], limit the expenditure of their remaining resources, and reduce behaviour that requires the consumption of resources [35,38], such as reducing the behaviour of value co-creation. Hence, the following is hypothesized.

Hypothesis 1 Customer incivility is negatively associated with the value co-creation behaviour of peer service providers.

#### 2.2.2 The mediating role of emotional exhaustion

The loss priority hypothesis of COR theory points out that long-term experience with low resources and high demand will lead to the loss of other resources [40]. In an environment with limited opportunities for resource renewal, long-term work stress will lead to burnout [41], one of the key factors of which is regarded as emotional exhaustion [42]. Emotional exhaustion occurs when individuals feel depressed under the influence of stressors, consider that they are short on the emotional resources to complete the required tasks, or feel or fear that their emotional resources are exhausted or about to be exhausted [43,44].

This research attempts to explain the relationship between customer incivility and the emotional exhaustion of PSPs from the perspective of COR theory. In the sharing economy, by investing surplus resources to cooperate with customers, solve their problems, and meet their service needs, PSPs can obtain some important resources (e.g., friendship and a sense of relevance) [45]. Therefore, in conventional value co-creation, it is not difficult for PSPs to achieve a balance between resource loss and resource acquisition. However, when customer incivility occurs, more requirements are placed on the emotional resources invested by PSPs. Simultaneously, the way to recover potential resources through efficient and pleasant interactions with customers may be cut off, which will aggravate the depletion of emotional or psychological resources and ultimately lead to the emotional exhaustion of PSPs. Therefore, the following is hypothesized.

Hypothesis 2 Customer incivility is positively associated with the emotional exhaustion of peer service providers.

As a type of resource investment, value co-creation requires users to input resources continuously [13]. In terms of COR theory, when individuals feel the loss or depletion of resources, they will be more inclined to protect and save the remaining resources they have [35,38] and reduce the input of existing resources [39]. Emotional exhaustion marks the depletion of emotional resources. When PSPs are in a state of resource exhaustion, out of the instinct to protect and conserve their existing resources, they will limit the expenditure of remaining resources and reduce value co-creation. Hence, the following hypothesis is put forward.

Hypothesis 3 The emotional exhaustion of peer service providers is negatively associated with value co-creation behaviour.

Based on these arguments, a mediating role of emotional exhaustion is predicted. Customer incivility reduces the value co-creation behaviour of PSPs due to increased emotional exhaustion. Customer incivility will cause the loss of emotional resources and excessive fatigue and may even lead to the emotional exhaustion of PSPs. To control the sense of resource exhaustion and avoid further resource loss, PSPs in the emotional exhaustion state will save their existing resources, reduce their resource expenditure, and reduce their value co-creation behaviour. Hence, the following is hypothesized.

Hypothesis 4 The emotional exhaustion of peer service providers mediates the association between customer incivility and value co-creation behaviour.

#### 2.2.3 The moderating role of self-efficacy for negative emotion regulation

Self-efficacy is a person's belief in whether he or she has the skills and resources necessary to succeed in a specific task or achieve a certain performance [46]. Persons with higher self-efficacy have stronger faith in completing tasks (including emotional work), investing more, and handling failure experiences better [47,48].

SENER refers to a person's belief in his or her capability to adjust and alleviate the negative emotions caused by difficulties and unpleasant situations [49]. Previous research has demonstrated that regulatory emotional self-efficacy regulates the association between emotional work needs and emotional disorders [50]. In the sharing economy, for PSPs with different SENER, due to their differences in the perception of emotional regulation ability, there are differences in their cognition and responses to the emotional resource loss caused by customer incivility. Specifically, PSPs with higher SENER hold stronger beliefs in their ability to regulate and ease adverse emotions. Once they perceive the loss of emotional resources caused by customer incivility, they will invest more to regulate negative emotions, reduce the loss of emotional resources, avoid a loss spiral, and ultimately decrease the occurrence of emotional exhaustion. Thus, the following is hypothesized.

Hypothesis 5 Self-efficacy for negative emotion regulation moderates the relationship between customer incivility and the emotional exhaustion of peer service providers. The relationship is stronger for peer service providers with lower self-efficacy for negative emotion regulation.

According to the preceding discussion, it can be further considered that SENER will moderate the indirect impact of customer incivility on the value co-creation of PSPs via emotional exhaustion. For PSPs with lower SENER, the indirect relationship will be stronger. Thus, the following is hypothesized.

Hypothesis 6 Self-efficacy for negative emotion regulation moderates the indirect relationship between customer incivility and the value co-creation of peer service providers via emotional exhaustion. The indirect relationship is stronger for peer service providers with lower self-efficacy for negative emotion regulation.

## 3 Research overview

The proposal was tested through three studies. In Study 1, online questionnaires were sent to PSPs in various fields of the sharing economy (including ride-share drivers, landlords of shared accommodation, and other providers of shared services). Based on the data gathered in three stages, the relationships between the variables in the research model were tested. However, due to the inadequacy of the questionnaire, a causal inference could not effectively be made. Therefore, the experimental method was used for further investigation. In Study 2, 100 PSPs from China (including ride-share drivers, landlords of shared accommodation, and other providers of shared services) were invited to participate in an online experiment. By manipulating the customer incivility received by the PSPs, the main effect (Hypothesis 1) was tested again, and the mediating role of emotional exhaustion in the relationship between customer incivility and the value co-creation of PSPs (Hypotheses 2, 3, 4) was verified. To further test the moderating role of SENER and enrich the representativeness of the research, samples from the United States were used for further tests. Study 3 focused on a common field of the sharing economy, namely ride-sharing. With the help of Amazon Mechanical Turk, 138 ride-share drivers from the United States participated in the online experiment. By manipulating the customer incivility received by ride-share drivers, Study 3 effectively examined the moderating role of the SENER of PSPs (Hypotheses 5, 6) and provided further support for the conclusions obtained in Study 1 (all the hypotheses) and Study 2 (Hypotheses 1, 2, 3, 4).

## 4 Study 1

### 4.1 Participants and procedure

To minimize the impact of common method bias, data were gathered in three stages from February 2022 to April 2022, with an interval of two weeks for each round of data collection. In each stage of the survey, multiple attention test items were set in the questionnaire to control the effectiveness of the response. If participants failed to pass these items, their questionnaires were rejected outright. In the first round of data collection (T1), 388 PSPs provided their demographic information and scored the customer incivility they perceived by filling out an online questionnaire. After excluding 38 invalid questionnaires, 350 valid questionnaires were retained (valid response rate: 90.21%). After two weeks (T2), a second online questionnaire was sent to the 350 valid respondents from the T1 survey, who were asked to evaluate their SENER and emotional exhaustion. In total, 265 questionnaires were gathered, among which 258 were valid (valid response rate: 73.71%). The third round of data collection (T3) was conducted two weeks after T2. Questionnaires were sent to those 258 participants who filled out the previous two questionnaires with valid results and who were asked to evaluate their value co-creation behaviour. A total of 220 questionnaires were returned, among which 217 were valid (valid response rate: 84.11%).

Data analyses were conducted by SPSS 26, the PROCESS plug-in in SPSS, and Amos. To check for response bias, an independent samples *t*-test was conducted to compare whether there were systematic differences between the gender, age, education, and other control variables of participants who left the study after the three-stage survey and those whose responses were retained. The results indicate that there was no significant difference [51,52] between the two types of samples for each variable (*p* > 0.05).

### 4.2 Measures

The measures used in this study were all mature scales that have been published. A five-point scale (from “1” = “strongly disagree” to “5” = “strongly agree”) was used in the measurement of all items.

**Customer incivility (T1).** Customer incivility was assessed by six items (Cronbach's alpha = 0.96) adapted from the research of Cho et al. [53]. The original item “restaurant customers” was changed to “customers” to adapt to the current research situation. An example item is “Customers took out their anger on me.”

**Emotional exhaustion (T2).** Emotional exhaustion was assessed by four items (Cronbach's alpha = 0.92) adapted from the study of Moore [54]. An example item is “It is tiring for me to wake up every morning and have to face the whole day's work.”

**SENER (T2).** SENER was measured with seven items using a scale (Cronbach's alpha = 0.86) adapted from Wu [49]. An example item is “When others try to argue with me, I can keep calm and control my anger.”

**Value co-creation (T3).** Value co-creation behaviour was assessed with five items (Cronbach's alpha = 0.85) adapted from the research of Yi and Gong [55]. An example item is “I will encourage others to use the car-hailing platform where I work.”

**Control variables (T1).** Referring to previous research, six variables were controlled for, including the participants’ gender, age, education level, number of years of work in the sharing economy, types of services, and whether they provide services on multiple sharing economy platforms simultaneously [2,13].

### 4.3 Study 1 results

#### 4.3.1 Common method bias

Respondents were guaranteed that the raw data would not be shared on any public platform, and the anonymous submission method was adopted to ensure the personal privacy of the respondents, thus improving the willingness of the respondents to answer frankly and further alleviating the common method bias.

However, because the data on all variables were gathered from the same survey object, the data may have been influenced by common method bias. Thus, the single-factor test was used to ensure that there was no such bias [56]. Via exploratory factor analysis, the total variance was examined by a single-factor model, in which the maximum variance explained by the model was just 33.89% (less than 40%). This indicates no obvious common method bias [57,58].

To further eliminate the possibility of common method bias, the addition of a common method factor was adopted. The confirmatory factor analysis model M1 was constructed, after which a common method factor was added to build model M2. The comparison of the fit indexes of the two models revealed the following: △*X*^2^/df = 0.073, △RMSEA = 0.002, △RMR = −0.002, △CFI = 0.003, △TLI = −0.005 (*X*^2^/df: chi-square/degree of freedom; RMSEA: root-mean-square error of approximation; RMR: root-mean-square residual; CFI: comparative fit index; TLI: Tucker-Lewis Index). Because there was no significant improvement in the model after adding the common method factor, there was no significant common method bias [59].

#### 4.3.2 Confirmatory factor analysis

Confirmatory Factor Analysis (CFA) was used to test the validity and model fit indices. The results reported in Table 1 demonstrate that the fit indices of the four-factor model met the critical value requirements (*X*^2^/df = 2.15, RMSEA = 0.07, RMR = 0.04, CFI = 0.93, TLI = 0.92). This model was superior to other alternative models, which means that the four variables considered in this research were characterized by good discriminant validity. In addition, the correlation coefficients of customer incivility, emotional exhaustion, SENER, and value co-creation behaviour were less than the square root of their average variance extracted (AVE) (Table 2), indicating that the key variables considered in this study had good discriminant validity [60].

**Table 1:** Confirmatory factor analysis (Study 1).

Factor model	*χ*^2^/df	RMSEA	RMR	CFI	TLI
4-factor model (CI/EE/VCC/SENER)	2.15	0.07	0.04	0.93	0.92
3-factor model (CI/EE + VCC/SENER)	3.56	0.11	0.05	0.84	0.82
3-factor model (CI + VCC/EE/SENER)	4.17	0.12	0.06	0.80	0.78
3-factor model (CI + EE/SENER/VCC)	4.86	0.13	0.10	0.76	0.73
2-factor model (CI + EE + VCC/SENER)	6.68	0.16	0.11	0.64	0.61
1-factor model (CI + EE + VCC + SENER)	9.07	0.19	0.12	0.49	0.44

*Note.* CI = Customer incivility, EE = Emotional exhaustion, VCC = Value co-creation, SENER = Self-efficacy for negative emotion regulation.

**Table 2:** Descriptive and correlation analysis (Study 1).

Variables	Mean	SD	1	2	3	4
*Study 1: n =* 217						
1. Customer incivility	2.94	0.94	**0.89**			
2. Emotional exhaustion	2.69	0.88	.43**	**0.83**		
3. SENER	3.96	0.54	−.17*	−.27**	**0.72**	
4. Value co-creation	4.13	0.42	−.25**	−.45**	.35**	**0.76**

*Note.***The underlined bold font** indicates the square root of the corresponding variables' AVE. *p < 0.05, **p < 0.01, ***p < 0.001. SENER = Self-efficacy for negative emotion regulation.

#### 4.3.3 Descriptive and correlation analysis

The descriptive statistics of the participants (Table 2) demonstrate that comparatively more participants were male (144; 66.36%) in comparison to female participants (73; 33.64%), and most of the participants belonged to the age group of 25–36 years (168; 77.42%). Moreover, most participants had bachelor's degrees (129; 59.48%) and had work experience of six months to three years (128; 58.99%). Customer incivility was found to be significantly positively correlated with the emotional exhaustion of PSPs (r = 0.43, *p* < 0.01), and negatively correlated with the value co-creation behaviour of PSPs (r = −0.25, *p* < 0.01). The emotional exhaustion of PSPs was found to be significantly negatively correlated with their value co-creation behaviour (r = −0.45, *p* < 0.01). This provides a basis for subsequent research and analysis.

#### 4.3.4 Hypothesis testing

The statistics reported in Table 3 demonstrate that customer incivility is negatively related to the value co-creation behaviour of PSPs (B = −0.11, *p* < 0.01). Moreover, customer incivility is positively associated with the emotional exhaustion of PSPs (B = 0.39, *p* < 0.01). When customer incivility and emotional exhaustion were simultaneously included in the regression equation, customer incivility remained positively associated with emotional exhaustion (B = −0.21, *p* < 0.01), whereas the significant association between customer incivility and value co-creation behaviour disappeared. Therefore, emotional exhaustion fully mediates the relationship between customer incivility and the value co-creation behaviour of PSPs.

**Table 3:** Test of mediating and moderating role (Study 1).

Variables	Test of mediating role						Test of moderating role	
	Value co-creation		Emotional exhaustion		Value co-creation		Emotional exhaustion	
	B	SE	B	SE	B	SE	B	SE
Gender	0.05	0.06	−0.16	0.12	0.01	0.06	−0.19	0.11
Age	0.01	0.01	−0.01	0.01	0.01	0.00	−0.01	0.01
Education	0.05	0.04	0.05	0.08	0.06	0.04	0.08	0.08
Tenure	0.03	0.03	−0.17**	0.06	−0.00	0.03	−0.18**	0.06
Service type 1	−0.12	0.08	−0.13	0.15	−0.14*	0.07	−0.08	0.14
Service type 2	−0.03	0.09	−0.05	0.17	−0.04	0.08	−0.01	0.17
Service type 3	0.10	0.11	0.12	0.22	0.12	0.11	0.25	0.22
Multiple	0.00	0.06	−0.08	0.11	−0.01	0.05	−0.04	0.11
Customer incivility	−0.11***	0.03	0.39***	0.06	−0.03	0.03	0.36***	0.05
Emotional exhaustion					−0.21***	0.03		
SENER							−0.14*	0.06
Customer incivility*SENER							−0.14*	0.06
Constant	4.01***	0.22	2.43***	0.42	4.51***	0.22	3.33***	0.39
R^2^	0.12		0.27		0.25		0.31	
F	3.00**		8.31***		6.99***		8.45***	

*Note. n* = 217. Source: Survey data. *p < 0.05, **p < 0.01, ***p < 0.001. Gender: 0 = Male, 1 = Female. SENER = Self-efficacy for negative emotion regulation.

In addition, the PROCESS plug-in in SPSS was also used to further test the mediating role of emotional exhaustion. The results indicate that the mediating role of emotional exhaustion was significant (indirect effect = −0.08, SE = 0.02, 95%CI = [−0.12, −0.05]). However, the direct effect of customer incivility on the value co-creation behaviour of PSPs is insignificant (direct effect = −0.03, SE = 0.03, 95%CI = [−0.09, 0.03]), indicating that emotional exhaustion fully mediates the association between customer incivility and value co-creation behaviour of PSPs.

Before examining the moderating role of SENER, the independent variable (customer incivility) and moderating variable (SENER) were standardized to eliminate the influence of multicollinearity. The hierarchical regression results reported in Table 3 demonstrate that the interaction term significantly and positively affects the emotional exhaustion of PSPs (B = −0.14, SE = 0.06, *p* < 0.05), indicating the moderating role of the SENER of PSPs.

**Fig. 2:**
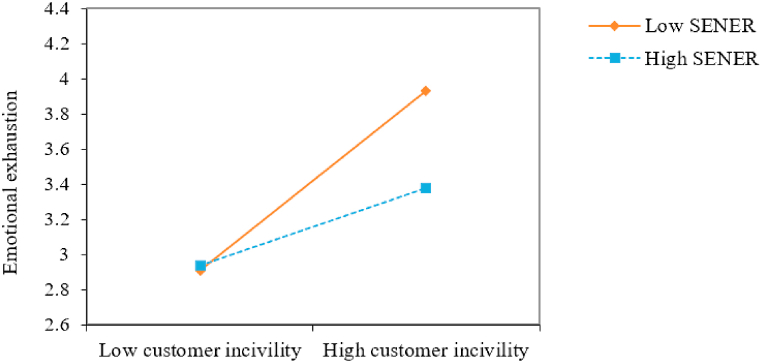
Study 1: Interaction of customer incivility and the self-efficacy for negative emotion regulation of peer service providers on the emotional exhaustion of peer service providers. *Note. n* = 217. The software developed by Dawnson and Richter [61] was adopted to plot the moderation effect. SENER = Self-efficacy for negative emotion regulation.

The interaction effect diagram was drawn by adding or subtracting one standard deviation from the mean value of SENER [61] to more intuitively reflect the moderating role of SENER (Fig. 2). It was found that when the SENER level is high, the association between customer incivility and the emotional exhaustion of PSPs is weak. Thus, SENER was found to buffer the mechanism by which customer incivility increases the chances of emotional exhaustion.

The final hypothesis explored the moderating role of SENER in the indirect relationship between customer incivility and the value co-creation behaviour of PSPs via emotional exhaustion. The PROCESS plug-in in SPSS was applied to test this moderated mediating relationship (i.e., OLS with interaction terms). The analysis results of Model 7 show that SENER regulates the adverse indirect effect of customer incivility on value co-creation via emotional exhaustion (indirect effect = 0.06, SE = 0.03, 95%CI = [0.01, 0.12]).

### 4.4 Summary

In Study 1, the relationships between the variables in the research model were tested by using data collected in three stages. The results showed that customer incivility in the sharing economy is positively correlated with the emotional exhaustion of PSPs, and the emotional exhaustion of PSPs is negatively correlated with their value co-creation behaviour. SENER was found to buffer the mechanism by which customer incivility increases the chances of emotional exhaustion. For PSPs with higher SENER, such relationships are weaker.

However, based on the results of the questionnaire alone, causal inferences cannot effectively be made, and the bias caused by poor memory cannot be avoided. Moreover, although the questionnaire was distributed in three stages with an interval of two weeks, which may have reduced some endogeneity problems, endogeneity cannot be eliminated entirely. In addition, due to the lack of data on the demographic information of the employees of the sharing economy in the Chinese market, the representativeness of the sample cannot be verified. Therefore, in Studies 2 and 3, experiments were conducted to further verify the research hypotheses.

## 5 Study 2

### 5.1 Participants and procedure

#### 5.1.1 Sample

The experiment was completed through the online platform Credemo, which allows for the random assignment of participants to different groups for manipulation. To improve the seriousness of the participants, each participant was told that if their answers passed the screening, they would be given a monetary reward. After the screening of an attention item, 100 PSPs (*M*_*age*_ = 33.09, 61.00% female) finally completed the experiment.

#### 5.1.2 Procedure

**Fig. 3:**
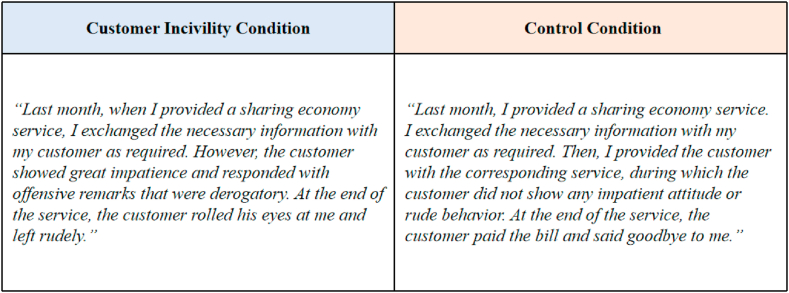
Study 2: Texts given to the participants in different conditions.

Participants were randomly assigned to the customer incivility condition (*n* = 50) or the control condition (*n* = 50). They were asked to read a text about a specific situation (Fig. 3) and put themselves in the person's shoes.

After reading the context, all participants were asked to fill out a questionnaire, which included manipulation checks, mediators (i.e., emotional exhaustion), the dependent variable (value co-creation behaviour), and demographic information. Multiple attention test items were set in the questionnaire to control the effectiveness of the responses. If the participants failed to pass these items, their answers were rejected outright. Moreover, a minimum display time was set for the pages that participants needed to read or fill in. They could click on the next question or turn the page only after the minimum display time was reached.

#### 5.1.3 Manipulation checks

A scale adapted from the research of Cho et al. [53] (Cronbach's alpha = 0.96) was used to evaluate the effectiveness of the manipulation. The results of the analysis of variance (ANOVA) test were *M*_*control*_ = 1.74, *M*_*incivility*_ = 3.97, F [1, 98] = 211.34, and *p* < 0.01, which proves that under the customer incivility condition, the customer incivility perceived by the PSPs was significantly higher than that under the control condition.

### 5.2 Measures

Participants scored the research variables on a five-point (from “1" = “strongly disagree” to “5" = “strongly agree”) scale based on the situations they encountered in the experiment. To assess their emotional exhaustion (Cronbach's alpha = 0.88) and value co-creation behaviour (Cronbach's alpha = 0.72), the same measures as those used in Study 1 were adopted.

### 5.3 Study 2 results

ANOVA was used to test whether the emotional exhaustion and value co-creation behaviour of PSPs are related to the customer incivility they receive. The results reveal that compared with the participants under the control condition, the participants under the customer incivility condition showed higher emotional exhaustion (F [1, 98] = 25.42, *p* < 0.01, *M*_*control*_ = 2.48 vs. *M*_*incivility*_ = 3.42), which supports Hypothesis 2.

The impact of customer incivility on the value co-creation of PSPs was then examined. The ANOVA results show that the value co-creation behaviour of the PSPs under the customer incivility condition was significantly lower than that under the control condition (F [1, 98] = 12.54, *p* < 0.01, *M*_*control*_ = 4.24 vs. *M*_*incivility*_ = 3.92), which supports Hypothesis 1.

Subsequently, the PROCESS plug-in in SPSS was used to examine the mediating role of emotional exhaustion in the impact of customer incivility on the value co-creation of PSPs. The results indicate that the mediating role of emotional exhaustion was significant (indirect effect = −0.13, SE = 0.05, 95%CI = [−0.25, −0.03]). Regarding the direct effect of customer incivility on the value co-creation behaviour of PSPs, direct effect = −0.19, SE = 0.10, 95%CI = [−0.38, 0.01], including 0, indicating a fully mediating role of emotional exhaustion. Thus, Hypothesis 4 is supported.

### 5.4 Summary

In Study 2, the main effect (Hypothesis 1) was tested again, and the mediating role of emotional exhaustion in the relationship between customer incivility and the value co-creation of PSPs (Hypotheses 2, 3, 4) was verified by manipulating the customer incivility received by the PSPs.

## 6 Study 3

### 6.1 Participants and procedure

#### 6.1.1 Sample

Through the online questionnaire platform Amazon Mechanical Turk, ride-share drivers from the United States were recruited to participate in this experiment. They were promised a reward of $0.20 to improve their participation and seriousness. After the screening of an attention item, 138 ride-share drivers (*M*_*age*_ = 35.25, 36.23% female) finally completed the experiment.

#### 6.1.2 Procedure

**Fig. 4:**
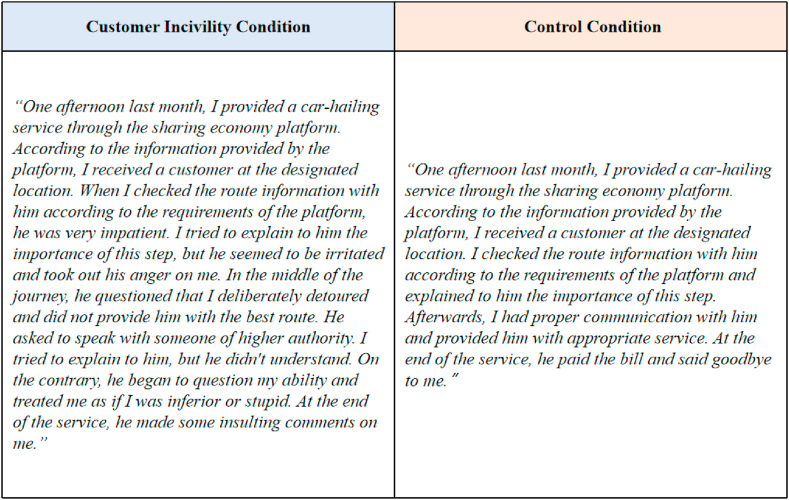
Study 3: Texts given to the participants in different conditions.

First, the ride-share drivers who participated in this experiment were asked to report their SENER. Participants were randomly assigned to the customer incivility condition (n = 71) or the control condition (n = 67). They were asked to read a text about a specific situation (Fig. 4) and put themselves in the person's shoes.

After reading the context, all participants were asked to fill out a questionnaire, which included manipulation checks, mediators (i.e., emotional exhaustion), the dependent variable (value co-creation behaviour), and demographic information. As in Study 2, multiple attention test items and a minimum display time were set to control the effectiveness of the responses.

#### 6.1.3 Manipulation checks

The same scale as that used in Study 2 (Cronbach's alpha = 0.93) was adopted to evaluate the effectiveness of the manipulation [53]. The results of the ANOVA test were *M*_*control*_ = 3.09, *M*_*incivility*_ = 4.17, F [1, 136] = 64.44, and *p* < 0.01, which proves that under the customer incivility condition, the customer incivility perceived by the PSPs was significantly higher than that under the control condition.

### 6.2 Measures

Participants scored the research variables on a five-point (from “1" = “strongly disagree” to “5" = “strongly agree”) scale based on the situations they encountered in the experiment. To assess the SENER (Cronbach's alpha = 0.85), emotional exhaustion (Cronbach's alpha = 0.88), and value co-creation behaviour (Cronbach's alpha = 0.82), the same measures as those used in Studies 1 and 2 were adopted.

### 6.3 Study 3 results

The ANOVA results support the positive correlation between customer incivility and the emotional exhaustion of PSPs (F [1, 136] = 56.73, p < 0.01, Mcontrol = 3.09 vs. Mincivility = 4.02) and the negative correlation between customer incivility and the value co-creation of PSPs. Thus, Hypotheses 1 and 2 are supported.

**Fig. 5:**
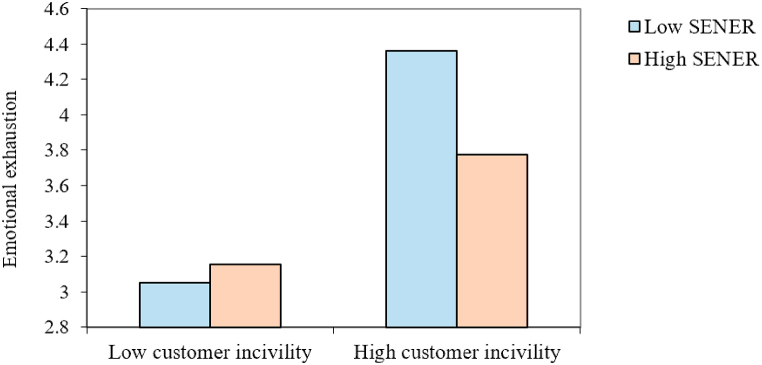
Study 3: Interaction of customer incivility and the self-efficacy for negative emotion regulation of peer service providers on the emotional exhaustion of peer service providers. *Note.* SENER = Self-efficacy for negative emotion regulation.

Next, the moderating role of SENER was examined. The regression results (Table 4) demonstrate that the interaction term constructed by customer incivility and SENER was negatively related to emotional exhaustion (B = −0.17, SE = 0.06, *p* < 0.01). The interaction effect diagram (Fig. 5) was drawn at high (+1 SD) and low (−1 SD) levels of SENER. It can be seen from the figure that when the SENER level was high, the positive impact of customer incivility on the emotional exhaustion of PSPs was weak, which supports Hypothesis 5.

**Table 4:** The results of regression (Study 3).

Variables	Emotional exhaustion	Value co-creation
**Independent variable**		
Customer incivility	0.96*** (0.12)	−0.16 (0.10)
**Moderator**		
SENER	−0.23 (0.12)	−0.08 (0.08)
**Interaction**		
Customer incivility*SENER	−0.17** (0.06)	−0.03 (0.04)
**Mediator**		
Emotional exhaustion		−0.36*** (0.06)
**R**^**2**^	0.35	0.37

*Note. n* = 138. Source: Experimental data. *p < 0.05, **p < 0.01, ***p < 0.001. Estimates are unstandardized regression coefficients. Values in brackets refer to standard errors (SEs). SENER = Self-efficacy for negative emotion regulation.

Finally, the moderating role was tested using the PROCESS plug-in in SPSS. The analysis results of Model 7 show that through the emotional exhaustion of PSPs, customer incivility was negatively related to the value co-creation of the PSPs (indirect effect = −0.33, SE = 0.08, 95%CI = [−0.49, −0.18]). Moreover, SENER was found to regulate the adverse indirect impact of customer incivility on value co-creation via emotional exhaustion (indirect effect = 0.22, SE = 0.10, 95%CI = [0.003, 0.41]). Thus, Hypotheses 4 and 6 are supported.

### 6.4 Summary

To further test the moderating role of SENER and enrich the representativeness of the research, samples from the United States were used for further testing in Study 3. Focused on a common field of sharing economy, namely ride-sharing, Study 3 effectively examined the moderating role of the SENER of PSPs (Hypotheses 5, 6) and provided further support for the conclusions obtained in Study 1 (all the hypotheses) and Study 2 (Hypotheses 1, 2, 3, 4).

## 7 Discussion and conclusions

The results of the three studies show that customer incivility is significantly negatively related to peer value creation behaviour and significantly positively related to emotional exhaustion. The emotional exhaustion of peer service providers mediates the association between customer incivility and value co-creation behaviour. Moreover, self-efficacy for negative emotion regulation was found to buffer the mechanism by which customer incivility increases the chances of emotional exhaustion. In accordance with the conservation of resources theory, this research extends the value co-creation literature and provides significant insights into the practice of platform management.

### 7.1 Theoretical implications

First, this research found a new antecedent that hinders PSPs from participating in value co-creation from the perspective of customer-provider interaction in the sharing economy. Prior research on value co-creation in the sharing economy has focused primarily on the value co-creation of consumers [2,12,13], while limited attention has been given to the value co-creation of PSPs [14]. However, in sharing economy services, the PSPs who provide the services and the customers who use these services form a bilateral market [15]. Therefore, the development strategy of sharing economy services obtained by analyzing only customers or PSPs is incomplete. This article discussed a new factor that affects the participation of PSPs in value co-creation from the perspective of customer-provider interaction, which further improves the relevant research on the influencing factors of value co-creation in the sharing economy. In addition, most previous research has shown that the factors of platforms and PSPs themselves are often important antecedents of the participation of PSPs in value co-creation in the sharing economy [22,25,26]. However, in the context of the sharing economy, some behaviours of customers may also hinder the participation of PSPs in value co-creation [21]. This research focused on identifying the antecedent of the value co-creation of PSPs in the sharing economy from the perspective of customers. By testing the new factors that affect the participation of PSPs in value co-creation (i.e., customer incivility), this research further expands the relevant research on value co-creation in the sharing economy.

Second, this research revealed the psychological mechanism underlying the association between customer incivility and the value co-creation of PSPs from the perspective of resource depletion and protection. The proposed mediating model is endorsed by COR theory, in which customer incivility in the sharing economy is taken as the independent variable, emotional exhaustion is taken as the intermediary variable, and value co-creation behaviour is taken as the dependent variable. The principle of COR theory proposes that the threat of resource loss results in the protection of existing resources. The findings of the present research provide evidence that customer incivility is perceived as a threat to valuable resources, thus leading to emotional exhaustion. If such pressures are not taken care of, they will lead to the reduction of value co-creation. Previous research has applied COR theory to explore value co-destruction [62], which is regarded as the opposite of value co-creation in the sharing economy [63]. The results of this research extend the application of COR theory to the research of value co-creation, explain the relationship between customer incivility and the value co-creation behaviour of PSPs from a new theoretical perspective, and further enrich the application context and scope of the theory.

Third, although previous studies have explored whether customer factors will have an impact on the value co-creation of PSPs [21], there have been few studies on the mitigation of this negative impact. The social cognitive theory of emotion regulation suggests that a person must believe that he or she can change his or her current emotional state (i.e., self-efficacy for emotion regulation) to successfully regulate negative emotional experiences [64]. Emotional regulation self-efficacy, as a self-efficacy with the quality of emotional regulation, is directly or indirectly related to the negative emotions of individuals [65,66]. Xu and Du [67] posited that emotional regulation self-efficacy may be an intrinsic mechanism of physical activity, which can improve adverse states such as depression and anxiety. Previous research has also shown that self-efficacy for emotion regulation regulates the association between emotional work needs and emotional disorders [50]. Therefore, this research introduced the SENER of PSPs as a moderating variable and analyzed the differences in the cognition and responses of PSPs with different SENER levels when facing emotional resource loss caused by customer incivility. The results of this research show that PSPs with a lower level of SENER often lack confidence in their ability to regulate negative emotions. When faced with the loss of emotional resources caused by customer incivility, they lack the motivation to mobilize resources to manage negative emotions, which further aggravates the loss and exhaustion of emotional or psychological resources, and ultimately increases the possibility of emotional exhaustion. This increases the flow of relevant documents and fills the gap of existing documents to a certain extent, and can help inhibit customer incivility in the sharing economy by enhancing the personality traits of PSPs to promote value co-creation in the platform ecosystem.

### 7.2 Practical implications

First, it was found that encountering customer incivility can reduce the value co-creation of PSPs. This suggests that platforms should be concerned about the relationship between the two types of users and actively take measures. For example, platforms can establish community guidelines and encourage users to abide by them. These guidelines can outline what is and is not acceptable behaviour on the platform, and what consequences users may face if they violate them. Furthermore, platforms can establish a customer support system to handle user complaints and reports of customer incivility. They should also have a system for responding to these complaints in a timely and effective manner. Platforms can also encourage bystanders to intervene when they witness customer incivility. This can be done by providing them with tools to report the behaviour or offering incentives for positive interventions.

Second, it was found that customer incivility reduces the value co-creation behaviour of PSPs by increasing their emotional exhaustion. This suggests that platforms should pay attention to the loss of users’ emotional resources, and measures should be taken to compensate for this loss, such as expanding the channels for frequent social interaction and offering opportunities for self-expression and emotional expression. Providing PSPs with timely measures to replenish their resources can help them better cope with the loss of resources due to interactions with customers, thus avoiding a spiral of losses.

Finally, it was proved that SENER weakens the positive correlation between customer incivility and emotional exhaustion. For PSPs with higher SENER, this positive correlation is weaker. Therefore, platform managers can take some measures to improve the SENER of PSPs. On the one hand, SENER can be included as an important reference index in assessing PSPs. PSPs whose SENER scores are lower than the threshold specified by the platforms should be required to participate in additional training courses to improve their emotion regulation ability; they can then officially start providing services only after they meet the basic requirements of the platform. On the other hand, psychological classes and training programs that can improve the SENER of users should be organized regularly.

### 7.3 Limitations and future research

This research was characterized by some limitations that can serve as extensions for future research. First, COR theory was drawn upon to investigate the mediating role of emotional exhaustion. In accordance with the theory, customer incivility will drain emotional and physical resources over time, causing a feeling of emotional exhaustion. To control the sense of resource exhaustion, PSPs will limit their expenditure and reduce the input of existing resources to ultimately reduce value co-creation. While COR theory provides effective support for this research, the dynamic process from perceiving resource depletion to limiting resource expenditure could not be directly examined. Although a three-stage longitudinal survey was adopted to collect data, some endogeneity problems remained. Future research can further overcome these possible problems via field experiments, instrumental variable testing, or other methods to clarify the impact mechanism.

Second, the research on value co-creation in the sharing economy has diversified theoretical logic and research ideas. In addition to the COR theory used in this research, the use of value co-creation theory within the service-dominant logic as the main theoretical lens is an important idea and direction that can be further explored in future research.

Third, the data collected in this research are only focused on Chinese and American samples. Although this could better ensure internal validity, there may be some deficiencies in the external validity and the universality of the research conclusions. In future research, PSPs in the sharing economy in multiple countries can be recruited for empirical testing, and more sufficient empirical evidence for the conclusion can be provided via the data testing of multiple national samples.

In addition, the standard deviation of value co-creation in Study 1 was relatively small, indicating that there may have been a limitation of little variation. Therefore, in Studies 2 and 3, the hypotheses proposed in this article were further tested using experimental methods.

## Funding

This work was supported by the 10.13039/501100001809National Natural Science Foundation of China (grant number 72102170, 72102172) and the Independent Research Project of Wuhan University (2021XWZY009).

## Production notes

### Author contribution statement

Yu Jia, Qinyu Chen: Conceived and designed the experiments; Performed the experiments; Analyzed and interpreted the data; Contributed reagents, materials, analysis tools or data; Wrote the paper.

Wenlong Mu: Conceived and designed the experiments; Analyzed and interpreted the data; Wrote the paper.

Wei Zhang: Analyzed and interpreted the data; Wrote the paper.

### Data availability statement

Data will be made available on request.

## Ethical statement

This study was conducted with the approval of the Human Subjects Ethics Sub-Committee of Wuhan University.

## Declaration of competing interest

The authors declare that they have no known competing financial interests or personal relationships that could have appeared to influence the work reported in this paper.
