# Supplementary material for: Managing the value Co-creation of peer service providers in the sharing economy: The perspective of customer incivility
Source: Heliyon. 2023 Jun 1;9(6):e16820. doi: 10.1016/j.heliyon.2023.e16820 (PMC10279822; doi:10.1016/j.heliyon.2023.e16820)
Supplement: Multimedia component 1 [file mmc1.docx]

| **Construct and sources** | **Description** |
| --- | --- |
| A five-point scale (from “1” = “strongly disagree” to “5” = “strongly agree”) was used in the measurement of all items. | |
| Customer incivility (Cho et al., 2016) | - Customers took out their anger on me. |
|  | - Customers made insulting comments on me. |
|  | - Customers treat me as if I was inferior or stupid. |
|  | - Customers showed that they are irritated or impatient. |
|  | - Customers did not trust the information that I gave them and ask to speak with someone of higher Authority. |
|  | - Customers made comments that question the competency of me. |
| Emotional exhaustion (Moore, 2000) | - I fell emotionally drained from the job of providing services on the sharing economy platforms. |
|  | - I feel fatigued when I get up in the morning and have to face another day at this job. |
|  | - I feel burned out from my job of providing services on the sharing economy platforms. |
|  | - I feel used up at the end of this work. |
| Self-efficacy for negative emotional regulation (Wu, 2020) | - When I encounter a difficult or unhappy situation, I can deal with my own feelings and resolve them by myself. |
|  | - When things go wrong for me, I can handle my emotions and don’t stay upset for long. |
|  | - When I am physically or emotionally hurt, I can process and handle painful emotions. |
|  | - When someone tries to start an argument with me, I can stay calm and control my anger. |
|  | - Even if I am treated unfairly, I can control my anger. |
|  | - When I need quiet and someone is bothering me, I can stay calm and control my anger. |
|  | - When I have planned a day and someone else messes up my plans, I can control my feelings of anger. |
| Value co-creation behavior (Yi & Gong, 2013) | - I help customers if they seem to have problems. |
|  | - If I have a useful idea on how to improve service, I will let the platform know. |
|  | - I will encourage others to use the car-hailing platform where I work. |
|  | - I will assist customers if they need my help. |
|  | - When customers do not appear at the agreed time and place, I would be patient. |
|  | - I will tolerate the occasional small mistakes of the platform. |
